# Sensory Survey 3D: an open source utility for the annotation of projected fields for sensory neural interfaces

**DOI:** 10.21203/rs.3.rs-9252777/v1

**Published:** 2026-04-01

**Authors:** Margaret Nielsen, Alexandriya Emonds, Mark Iskarous, Ali Alamri, Leah Roldan, Rohit Bose, Emily Graczyk, Charles Greenspon

**Affiliations:** University of Chicago; University of Chicago; University of Chicago; University of Chicago; Case Western Reserve University; Case Western Reserve University; Case Western Reserve University; University of Chicago

**Keywords:** projected fields, somatosensation, intracortical microstimulation, peripheral nerve stimulation, sensory restoration

## Abstract

**Background:**

One goal of neuroprosthetics is to restore sensation to those who have lost it due to disease or injury. This is often accomplished with electrical stimulation of nervous tissue. Common approaches to quantifying evoked sensations for somatosensory prostheses involve asking participants to draw or annotate flattened 2-dimensional (2D) representations of body parts, which can distort the 3-dimensional (3D) projected fields. In particular, patches of skin between the fingers and toes often go unrepresented in 2D, leaving them unquantified. Here, we present a 3D annotation tool that allows participants to accurately report evoked sensations on any given body part. Additionally, we present a pipeline for creating patient-specific models to accommodate different morphologies, and algorithms to synthesize data across models to facilitate analysis.

**Methods:**

Patients implanted with either NeuroPort electrode arrays (Blackrock Neurotech) in Brodmann’s Area 1 (N = 2 male) or Composite Flat Interface Nerve Electrodes on the peripheral nerves of an amputated arm (N = 2 male) were electrically stimulated and asked to annotate the location of the resulting sensations. In the first cohort, patients reported sensations on a generic hand using either a 2D or 3D interface. We computed the Jaccard index for each electrode between annotation methods and the proportion of 3D faces distorted during the 2D projection. In the second patient cohort, as proof of concept, we created a 3D model of each participant’s residual hand and then mirrored it, allowing participants to annotate their amputated hand. We then projected annotations of each custom model onto a generic model to enable direct comparison of annotations across participants.

**Results:**

We found that participants reported consistent sensation locations between annotation methods when sensations were localized to central parts of the fingers and hand, visible in 2-dimensions. However, as expected, the 3-dimensional interface better captured sensations on the edges of or between fingers. Development of an iterative Procrustes alignment algorithm allowed for easy comparison of projected fields between patient-specific models.

**Conclusion:**

Our 3-dimensional annotation tool allows for more precise annotation of evoked sensations than 2-dimensional alternatives and facilitates rapid analysis across patients with different morphologies.

## Introduction

**Figure 1:**
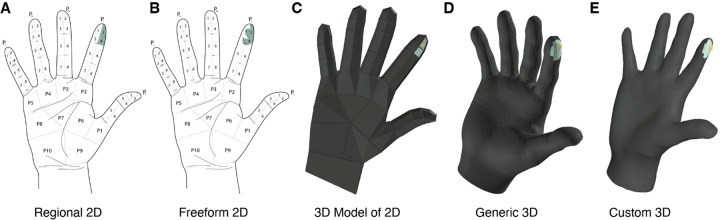
Old and new templates for projected field annotation. A| Example 2D segment level annotation of the palm where participants verbally report segments. Labels indicate (P)almar segment numbers, and which digit the segment corresponds to (thumb, index, middle, ring, or pinky). B| 2D freeform annotation of the palm, manually annotated with a stylus. C| 3D model of the annotation in A adapted for Sensory Survey 3D. Selected faces are shown in green. D| Survey 3D’s default generic model of a man’s right hand with a similar annotation. E| A participant’s custom 3D hand model created via photogrammetry with a similar annotation.

Sensory restoration is a longstanding goal of neural interfaces for those who have lost sensation due to injury or disease^1^. One solution is the direct electrical activation of either the brain or peripheral nerves^2^. To understand the nature of sensations evoked through electrical stimulation, participants are typically asked to report the location (often known as the projected field) and quality of these sensations. In the case of somatosensory research, this typically involves annotation of a flattened representation of the arm or leg in a 2D plane on a piece of paper or digital tablet^3–9^ (Fig. 1A, B). Projecting or flattening body diagrams simplifies reporting but can obfuscate the locations of sensations. As we work toward high-resolution sensory feedback^10,11^, accurate annotation of projected fields will be essential for optimal mapping of robotic sensors to electrodes. Moreover, current reporting methods typically involve annotating a generic diagram of the target limb or body part. Future state-of-the-art reporting methods must allow study teams to account for differences in patient body morphology by providing patient-specific body models for annotation.

While many groups record the projected fields for various stimulus modalities, no standardized system exists for recording these sensations. Leveraging over a decade of experience in delivery of artificial sensory feedback to hand knob–adjacent regions of human somatosensory cortex and the sensory peripheral nerves^6,8,12,13^, we identify several drawbacks to existing annotation approaches with generic, 2D body representations. First, flattened representations consistently lead to over or under-representation of certain parts of the body, such as the commonly omitted edges of fingers or fingertips as well as the side of the hand, leading to distortion of the reported projected similar to when a Mercator projection is used. This limitation is especially salient given the importance of fingertips in dexterity and manual interactions with objects in the world. Second, sensations can “wrap around” the body; individual electrodes often evoke a sensation that covers both the palmar and dorsal surface of the hand, forcing participants to create separate reports for the front and back of the hand. These unconnected reports lead to misinterpretation, either between the fingers or at the ulnar or radial extents of the hand. Thirdly, the use of generic models can obfuscate individual differences between subjects. In the case of amputees, patients often report sensations on the residual limb in addition to the phantom limb. As each amputation is different, there is no way to annotate the residual limb using a generic illustration, resulting in lost information. Furthermore, patient-specific diagrams of body parts could become increasingly important, as future applications of sensory neuroprostheses could involve sensory restoration on areas of the body with high variability across people, such as the face, chest, or torso^14^. Fourth and finally, projected fields do not always exhibit uniform intensities^5,15^, sometimes presenting as hotspots with diffuse borders, the location of which is similarly susceptible to distortion. To address these limitations, we created Sensory Survey 3D: an open-source application capable of comprehensive, accurate, and precise annotation of artificially evoked sensations on patient-specific 3D models of target body part(s). Moreover, we describe a pipeline for scanning residual limbs and importing custom meshes into the annotation tool, allowing participants to annotate accurate models of themselves. Finally, we implement several accessibility features to facilitate usage by a wide range of experimenters and patients.

Using a 3D interface requires reimagining how projected fields are conceptualized and analyzed. Typically, 2D projected fields are composed of binarized pixels or groups of pixels (often referred to as segments) and a body part can be annotated from multiple 2D views in which the pixels cannot be compared between views. With 3D models, the entire body part is composed of vertices and faces, all existing in the same space. In this work, we test our newly developed 3D annotation tool (Fig. 1C–E) against a previously developed 2D tool that we have used for ~ 5 years. We find that when comparing projected fields annotated in 2D or 3D, the 2D annotations failed to accurately capture size and shape of sensations near the edges of the illustration (those between fingers and on the sides of the hand).

## Methods

### Participants

Participants from two separate and ongoing clinical trials at the University of Chicago and Case Western Reserve University were included in this work. The participants at the University of Chicago were spinal cord injured patients implanted with two 32-channel Blackrock NeuroPort electrode arrays (Blackrock Neurotech, Salt Lake City, UT, USA) in somatosensory cortex. For detailed information on the participants and electrode implantation, see Downey 2024^16^. The participants at Case Western Reserve University were unilateral upper limb amputees implanted with 2–3 16-channel Composite Flat Interface Nerve Electrodes (C-FINE) on the residual median, radial, and/or ulnar nerves of the amputated arm. For details on the implant and system, see Lambrecht 2024^17^. See *ethics approval* for more information on the clinical trials.

## Survey software dependencies

The Sensory Survey 3D application consists of two components: 1) a web interface written in HTML and JavaScript and 2) a server backend written in Python. The web interface is the frontend through which participants report annotation data. Three.js (https://threejs.org/), an open-source 3D graphics library for JavaScript, was used for the rendering of 3D models and participant annotations. Annotations appear on the surface of the mesh through vertex coloring.

The server backend enables networked, cross-computer survey administration through HTTP and WebSocket communication. Percept data is streamed from an ongoing survey to the backend over WebSocket, preventing frontend errors from causing data loss and allowing the optional “Experimenter” page to display live updates of user input. FastAPI (https://fastapi.tiangolo.com/), an open-source web framework, was used to define HTTP and WebSocket behaviors for the backend. Uvicorn (https://uvicorn.dev/), an Asynchronous Server Gateway Interface web server implementation for Python, was used to run and broadcast the backend over the network.

### Annotation of projected fields in 2D and 3D

Participants at the University of Chicago were asked to annotate sensations on either the 2D interface we have previously used for recording responses^5^ (Fig. 1A, B) or the Sensory Survey 3D application (Fig. 1C–E). Electrodes (N = 33 and 55 for C1 and C2, respectively) were selected and presented in a random order to each of the participants. Annotation was performed in a blocked manner where participants annotated each electrode using either the 2D or 3D interface before the interface was swapped and all electrodes were annotated again. Electrode order was randomized within block. Data collection was performed across 8 sessions (5 for participant C1, 3 for participant C2) and the electrode order was again randomized for each. For each survey, stimulus parameters were adjusted such that a supra-threshold amplitude based on a 3D1U transformed staircase^18^ to find the threshold. All stimuli were 1 second in duration and delivered at 100 Hz. Participants were allowed to request as many repeated stimuli as desired for each survey. For both the 2D and 3D interfaces, participants used a touchscreen laptop with a stylus using residual movement capabilities. Experiments with participants at Case Western Reserve University are described below in *‘Residual limb scanning and reconstruction’*.

## Comparison of projected fields between 2D and 3D interfaces

To compare the 2D and 3D survey annotation data, we flattened and warped the 3D model such that faces perpendicular to the user’s perspective (fronto-parallel) matched those of the 2D reference hand. To achieve this, we implemented an iterative Procrustes procedure described below. Critically, these steps can be applied to any model and are not restricted to the hand. Most structures will be simpler than the hand, requiring fewer processing steps for analysis.

## Landmark definitions

We placed primary landmarks at the tips of the thumb, index, middle, ring, and pinky digits (5 landmarks); over the centers of the distal and proximal interphalangeal joints of each finger (8); over the center of the interphalangeal joint of the thumb (1); over the centers of the metacarpophalangeal joints of each digit (5); at the middle of the palm on the palmar surface (1); at the middle of the palm on the dorsal surface (1); and at the center of the base of the wrist (1). Accessory landmarks were taken as width markers for various parts of the hand. Width markers were selected at the dorsoventral midline halfway along each segment of each finger and the thumb (28). Accessory landmarks were also defined at the thumb-side and pinky-side junctions between the wrist and the palm (2) and across the widest area of the palm (2).

### Initial Procrustes alignment

The alignment between the 3D mesh and the palmar 2D illustration and the alignment between the 3D mesh and the dorsal 2D illustration were performed in series. In each case, we first calculated the translation, scale, and orthogonal rotation and reflection components of an unrestricted Procrustes alignment between the XYZ positions of all 54 landmarks on the 2D and 3D models, then applied the computed transformation. Finally, we translated the 3D hand model such that the anchor point, defined as the landmark at the end of the wrist, was identically positioned across both models.

## Dependency specification

We considered the hand as a hierarchical structure where adjacent segments must be attached to each other, then created a dependency list reflecting this. When aligning the 3D mesh to the 2D reference, it is not sufficient to flatten the Z-axis; instead, each segment must be rotated and scaled appropriately. However, before this rotation can be applied, it is essential to allocate each vertex of the 3D mesh to the correct segment. To determine which segment each vertex belonged to, we created an expanded landmark set by interpolating between primary landmarks as defined in the dependency list. We then assigned each vertex to the segment of the nearest temporary landmark, with partial influence (exponential falloff based on distance) from landmarks with linked dependency.

## Iterative segmented Procrustes procedure

Iterating through the list of dependencies (**Supp** Fig. 1), the algorithm:

1. Considered which primary landmarks were involved in the current dependency pairing.
2. Expanded the list of landmarks to be used for segmented Procrustes alignment to include those with similar base names (e.g. *Rdip* to *Rdip_t* and *Rdip_p*) within the set of accessory landmarks.
3. Computed the translation component, scale component, and orthogonal rotation component (excluding the possibility of reflection) of the Procrustes alignment between the superset of relevant primary and accessory landmarks.
4. Applied these components to the superset of relevant primary and accessory landmarks as well as all vertices controllable by the superset (as described in Dependency specification, above).

### Rasterization of 3D faces into 2D pixels and obliqueness calculation

After the 3D mesh and landmarks were morphed to align with the 2D landmarks, we partitioned the faces of the 3D mesh into palmar and dorsal planar aspects according to their normal vectors (**Supp** Fig. 2A, 0° to 90° and 90° to 180° from the depth vector, respectively). A third category of faces, oblique faces, with normals greater than 60° and less than 120° from the camera or depth vector, were also identified, though oblique faces were not excluded from the palmar or dorsal categories (**Supp** Fig. 2B). We then separated the palmar and dorsal vertices into individual palmar and dorsal meshes that were flattened for direct comparison with 2D annotation maps.

### Overlap between 2D and 3D annotations after rasterization

Once rasterized, the 3D annotations could be directly compared with the 2D illustrations. We used the Jaccard index to estimate the proportion of overlap between two annotations. The Jaccard index compares the intersection of the two annotations with the total area (union) of both annotations together:

$$J=\frac{2D\cap 3D}{2D\cup 3D}$$

### Generic 3D body models

A set of pre-partitioned, re-topologized body parts derived from the Blender Foundation’s free Human Base Meshes v1.4.0 asset bundle (https://www.blender.org/download/demo-files/#assets) is available for immediate use with the Sensory Survey 3D application via the SensorySurvey3D repository on GitHub.

### Residual limb scanning and reconstruction

The residual arms and hands of the participants at Case Western Reserve University were scanned using the EM3D scanning application (Ethan Makes, https://em3dscanningapp.com/) on an Apple iPhone. Outliers in the resultant point clouds were automatically removed in RevoScan5 (https://revopoint3d.com). OBJ meshes were exported from RevoScan5 and imported into Blender (https://www.blender.org/), where any additional artifacts (e.g. non-manifold geometries) were manually resolved. OBJs were re-meshed using the Instant Meshes^19^ utility (Wenzel Jakob, https://github.com/wjakob/instant-meshes), uniformizing face sizes for ease of annotation. Finally, using Blender, OBJs were reflected across the midline to represent the contralateral amputated limbs and exported as GLB files compatible with the Sensory Survey 3D tool. Participants were then asked to complete 3D surveys while PNS was delivered to individual contacts (N = 4) in the C-FINEs. Stimulus parameters and contacts varied for each survey, again ensuring that supra-threshold stimuli were delivered.

### Procrustes transformation between 3D models

The procedure for alignment of landmarks between two 3D models was the same as the procedure for alignment of landmarks between one 3D mesh and one 2D illustration, as described above. To morph from one 3D mesh to another 3D mesh, one must be defined as the “source” and the other the “target” (Supp Fig. 3A-C). Both models must have matching sets of landmarks defined. In the case of 3D-to-3D transformation, the patient-specific model would typically be labelled as the source and the generic model the target, such that all patient-specific models get warped to the common generic model. In the case of warping a 3D mesh to a 2D illustration, the source landmarks are landmarks of the 3D mesh while the target landmarks are those of the 2D illustration.

Additionally, a sparse transfer matrix quantifying the projection of proximal faces of the source mesh onto each face of the target mesh along the target face’s normal vector is computed once for each pair of source and target meshes. We constructed a k-dimensional (k-d) tree out of source mesh face XYZ locations. For each face in the target mesh (**Supp** Fig. 3D, red face), we performed a k-nearest neighbors search to identify the k = 30 nearest faces in the source mesh by Euclidean distance (**Supp** Fig. 3E, blue faces). Importantly, the value of k should scale with the ratio of source mesh face count to target mesh face count; our ratio was approximately 1:1. After parsing the locations of the target face vertices and the source face vertices for all 30 nearest source faces, we subtracted the target face location from all source and target vertices to re-zero them. We calculated the rotation matrix necessary to rotate the target face’s normal vector to a unit vector pointing along the Z axis and multiplied the vertex locations of the target face and the vertex locations of all source faces by that matrix. We then zeroed the Z component of all source vertices to produce a perspective view of source faces along the target face normal (**Supp** Fig. 3F). In this 2D planar projection, we computed the area of overlap of each source face with the target face and divided by the total area of the target face. We stored the proportional coverage of each flattened source face in the transfer matrix at position (target vertex, source vertex) for all combinations of target face and relevant source face vertices.

To project an arbitrary annotation of the source mesh onto the target mesh, we multiplied the source mesh’s projected field array by each row of the transfer matrix. We then summed over columns to form a new projected field array with the same number of rows as the number of vertices in the target mesh. Values in the new projected field array that were greater than or equal to 1.5 (3 vertices per face × 50% coverage) were set equal to 1, annotating the corresponding faces. Values less than 1.5 were zeroed.

## Results

### Overview of the Sensory Survey 3D’s features

**Figure 2:**
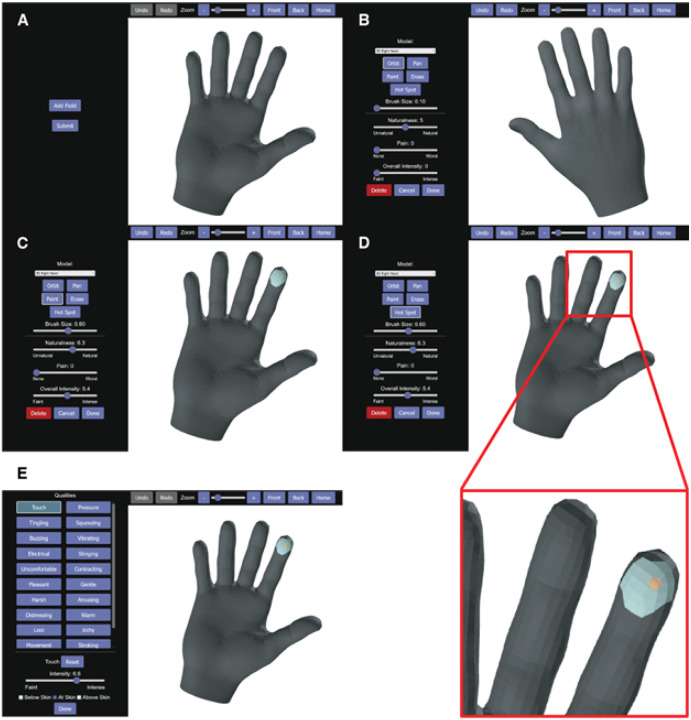
Sensory Survey 3D usage and features. **A|** Participant home screen, displaying the selected mesh from those specified in the participant configuration file (“config”). **B|** In addition to “Orbit” and “Pan” tools for navigation, default view options “Front” and “Back” for a given mesh are stipulated in the config. **C|** “Paint” produces a brush of customizable size. Clicking on and dragging across the model’s faces edits the active annotation colormap. Mistakes can be amended with the “Erase”, “Undo”, and “Redo” tools. **D|** The most intense sensation or point of sensation origin can be documented using the “Hot Spot” tool. Following placement, hot spots are visible on the mesh surface as orange spheres (see inset). **E|** Any number of commonly experienced sensation qualities can be provided as annotation label options within the config. Prior to finalizing the annotation details, quality is assigned an intensity rating and an at-, above-, or below-skin locale by the user.

Upon launching the application, the participant is presented with a home screen displaying a default mesh of choice (Fig. 2A), as specified in the participant configuration file. Once stimulation has begun, the participant can annotate a new projected field map by selecting “Add Field.” After choosing a mesh from the “Model” drop-down menu, the participant can rotate and translate the mesh within the application to gain access to self-occluded areas of the model with the “Orbit” and “Pan” tools, respectively. Frequently used perspectives can be defined (e.g. “Front” and “Back”) in the participant configuration file to create shortcuts (Fig. 2B). Selecting “Paint” produces a brush of customizable size, and clicking on and/or dragging across faces changes their color within the application and assigns them to the active annotation colormap (Fig. 2C). Faces can be removed from the active annotation colormap with the “Erase” tool. Participants can mark one or more specific points of interest within the annotation map—areas of intense sensation or points of origin for a given sensation—using the “Hot Spot” tool (Fig. 2D), which places an orange sphere at the relevant locus. Projected field annotations can be scored on a “Naturalness” visual analog scale (from unnatural to natural), a “Pain” scale (from none to worst), and an “Overall Intensity” scale (from faint to intense) prior to finalizing with the “Done” button, which brings up a menu of “Qualities” to associate with the annotation (Fig. 2E). Multiple qualities can be added for each annotation, each given its own “intensity” and “depth” (above, at, or below the skin). The list of available qualities is customized in the participant configuration file such that per-participant quality descriptor lists can be customized or held constant, as desired.

### Segmented Procrustes can be used to compare responses across 2D and 3D models

**Figure 3:**
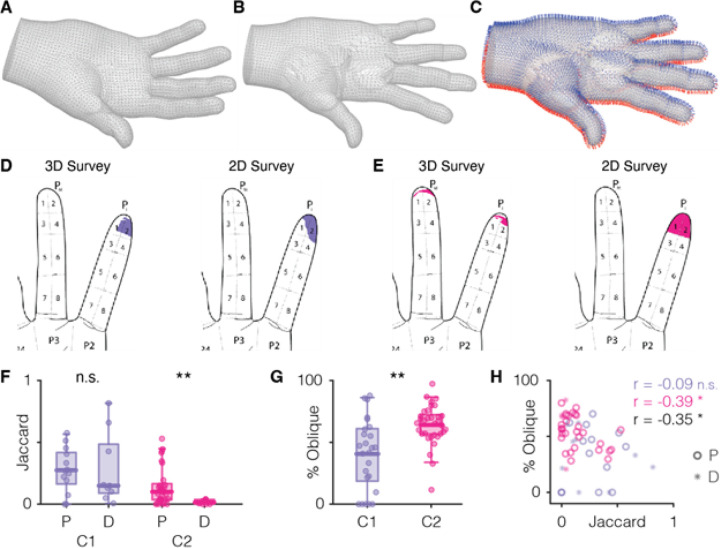
Quantifying differences between electrode-matched 2D and 3D surveys. A| View of Survey 3D’s default right hand model before Procrustes adjustments. B| A, following the iterative segmented Procrustes procedure. C| B, annotated with face normals color-coded as palmar (blue) or dorsal (red). D| Example 3D survey projection onto 2D palmar illustration for C1 (left) compared with the 2D survey for the same electrode (right). JI = 0.58, obliqueness = 41%. E| Example pair for participant C2, JI = 0.12, obliqueness = 70%. F| Distribution of Jaccard indices for (P)almar and (D)orsal annotations across electrode, separated by participant. C1 P N=13, D N=9; C2 P N=29, D N=6. G| Distribution of obliqueness scores of 3D surveys for participants C1 (N=25) and C2 (N=33). H| Scatter plot comparing the Jaccard indices with obliqueness scores. Palmar map comparisons are represented by open circles and dorsal map comparisons are represented by stars. Pearson correlation coefficients for C1’s maps only (purple), C2’s maps only (pink), and the combined set of maps for both participants (black) are also reported.

Two participants with SCI completed 3D surveys using the Sensory Survey 3D default male right hand model (Fig. 1D, Fig. 3A). To quantify differences in projected field annotation maps produced with the 2D and 3D interfaces, we first had to morph the vertices and faces of the 3D model such that morphological features including palm width, digit joint length, and digit adduction/abduction and flexion/extension were matched across the 3D model and the palmar and dorsal 2D illustrations. We accomplished this with an iterative segmented Procrustes algorithm (see Methods, **Supp** Fig. 1). Our morphed 3D model (Fig. 3B) was then separated into dorsal and palmar aspects based on face normal vectors (Fig. 3C) and flattened into constituent planar dorsal and palmar meshes (**Supp** Fig. 2A) for direct comparison to 2D annotation maps.

#### 3D annotation of body parts enables precise localization of sensations

To compare the reported sensations, we computed the Jaccard index for pairs of 2D and flattened 3D annotations for each tested electrode, where 0 indicates no overlap and 1 indicates perfect correspondence between the annotations. As expected, we found that the Jaccard index varied depending on annotation location. When projected fields near the center of a finger pad and annotated faces were perpendicular to the user, the Jaccard index was high between 2D and 3D representations of the projected field of the same electrodes (e.g. Figure 3D, JI = 0.58). When projected fields were near an edge of the 2D diagram and thus the 3D faces were oblique, the Jaccard index was much lower (e.g. Figure 3E, JI = 0.12). Jaccard indices varied widely for each participant (Fig. 3F, C1: 0.28 ± 0.23, C2: 0.12 ± 0.15), and in the case of C2, they were higher for electrodes with projected fields on the palmar surface of the hand than those on the dorsal surface (Wilcoxon rank sum test, p < 0.01). This effect was not driven by biases in electrode selection between participants (**Supp** Fig. 4). While the alignment and warping procedure could lead to misalignments in the data, we believe that these differences reflect both variation in user reporting^5^ and true differences in estimation as a result of 2D versus 3D perspectives.

We hypothesized that a major source for discrepancies between classic 2D and flattened 3D annotations would be distortions at the periphery of the 2D illustrations around the edges of the digits and hand. Sensory Survey 3D eliminates this problem, but large swathes of annotation area are obscured in flattened 3D annotation maps as a result (e.g., Fig. 3E). To quantify this, we calculated the proportion of oblique surface area in each 3D survey annotation (**Supp** Fig. 2B, Fig. 3G). We found that both C1 and C2 had average obliqueness scores above 40% (41% ± 29% and 64% ± 16%, respectively). Moreover, C2’s 3D surveys were significantly more oblique than C1’s (Wilcoxon rank sum test, p < 0.01), perhaps explaining the smaller Jaccard indices reported for that participant, though it also possible that this is due to the less precise segment-level annotation tool that C2 used. We computed the Pearson correlation between obliqueness and Jaccard index both within and across participants and found a significant negative correlation for the combined set of surveys from C1 and C2 (p = 0.0214 after Bonferroni correction) and for C2 alone (p = 0.0424), but not for C1 individually (Fig. 3H, p > 0.05 after Bonferroni correction).

#### 3D scanning of participants’ body parts for more intuitive and accurate labelling of sensations

**Figure 4:**
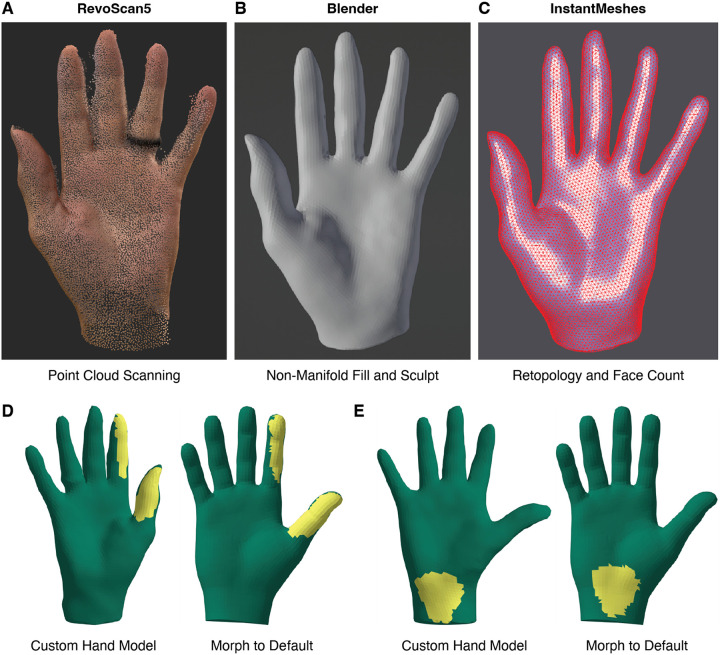
Pipeline for incorporation of custom 3D models. A| Point cloud produced through EM 3D photogrammetry using an Apple iPhone, following RevoScan5-automated outlier removal. The left hand of a participant with right transradial amputation was scanned. B| RevoScan5-exported mesh in Blender following verticalization, resizing, manual filling of non-manifold geometry (holes), and creasing and smoothness sculpt. C| Retopology and rough face count specification via InstantMeshes. The finished mesh was exported from InstantMeshes as an .obj, imported into Blender, reflected, and re-exported in .glb format for use with Sensory Survey 3D. D| The amputee participant annotated a mirrored right-hand version of their custom model during peripheral nerve stimulation. Example morph of custom 3D mesh to Survey3D’s default right hand model. The projected field map (yellow) is retained for direct comparison of annotations across models. E| Another example projected field, with a different starting custom 3D mesh (see Figure 1E).

While generic body parts can be used for consistent annotations, substantial variation can occur between participants. This requires that participants use vague internal heuristics (that may not be shared across participants) to warp their perception of sensations on their own body to the generic body parts they are shown. To address this, custom 3D models can be used with Sensory Survey 3D in addition to the generic models distributed with the application. A point cloud of a participant’s hand can be generated using any number of photogrammetry tools (Fig. 4A), processed with mesh sculpting and modification software (such as Blender, Fig. 4B), and retopologized to the desired face count (e.g. with InstantMeshes, Fig. 4C), before it is converted to a Sensory Survey 3D-compatible mesh. A full pipeline for this procedure is provided on GitHub. This custom mesh approach has particular value for amputees, where the level of amputation is highly variable and sensations might be expected on the residual limb topology that cannot be easily captured with default models. As a proof of concept, we scanned the non-amputated limbs of two participants with unilateral upper limb amputation, mirrored them, and had the participants annotate their own limb model. Crucially, software distributed with the Sensory Survey 3D application enables the projection of annotations on custom 3D models onto a stereotyped mesh for direct comparison across participants (see Methods for details). Examples of morphs to a generic hand mesh are presented for custom scans of these amputee participants in Fig. 4D and Fig. 4E, illustrating the reliability and accuracy of annotation projections to a standard model.

## Discussion

### 3-dimensional models allow more precise annotation of projected fields

Mapping 3-dimensional structures to 2-dimensional planes is a problem across a range of domains. Mapping sensations to the front or back of the hand may be trivial, but the fingertips, edges of the fingers, or sides of the hands can be difficult. Moreover, feature-poor body parts such as the arm or leg can be even more difficult to accurately report on 2D diagrams, as participants have few visual landmarks for orientation. Our results comparing 2D and 3D annotations confirm this issue and demonstrate that the usage of 3D annotation tools can minimize these problems by allowing participants to interact with realistic models. We believe that the results from the 2D versus 3D comparisons suggest that annotating on a custom 3D body model will yield even higher accuracy, though we explicitly only show this as a proof of concept application. We believe that the ability to annotate custom models will be more accurate and easier for participants, since it offloads scaling and warping to robust algorithms instead of requiring participants to mentally apply these transformations themselves, a procedure that is unlikely to be consistent even within a single session.

### Further uses of the application

While both patient cohorts evaluated in this study were enrolled in clinical trials that focused on sensory restoration to the upper limb, efforts to restore sensation are not limited to that area. Indeed, neuroprosthetics for lower limb amputees are another growing field^20^, and recent efforts have been made to work towards the development of a prosthetic breast that could restore sensation^14,21^. As sensory restoration grows beyond the hand, tools that can be flexibly used across any body part and analyses that allow for participant-specific annotations will become increasingly important. While hands and feet may be considered relatively stereotyped, breasts are vastly more heterogeneous and standardized models may not be suitable for annotation^21^. Finally, in several cases, nerve stimulation results in sensory percepts on the residual limbs of amputees^20,22,23^, which much like breasts, can vary substantially depending on the nature of the injury and the surgical intervention. Both the scanning pipeline detailed here and the tools for converting between models will thus have broad applications across patient populations and sensory restoration technologies.

Beyond sensory restoration, this tool provides a standardized way to report or annotate any kind of sensation or injury. From reporting pain at a single time point to tracking the progression of an intervention over time, digital representations with high accuracy, that enable reporting of complex sensory characteristics, and amenable to systematic comparison across patients could allow for greater understanding of disease progression or clinical interventions. The ability to compare across models also eliminates the need for data synthesis across studies and teams to require utilization of the same model, further facilitating collaboration. Sensory Survey 3D is distributed with a rudimentary MATLAB-based annotation viewer to render stored annotations, enabling easy viewing of the maps it creates (**Supp Fig. 6**), as well as a set of functions to perform the morphs and iterative transformations described in this report, making it a highly adoptable resource for researchers in a variety of fields.

### Considerations for ease of use across patient populations

Given the broad range of patient populations to which this tool might apply, it is worth considering how it might be utilized differently by different groups. The Sensory Survey 3D interface is modular and scalable in complexity, enabling data collection from participants with a wide range of physical and cognitive capabilities. For example, in cases of spinal cord injury or upper limb amputation, patients may lack fine motor control required for precise annotation; by contrast, when the upper limbs are unaffected, patients may be able to provide extremely detailed annotations. The neural interface that the patient has received is also relevant. Broadly, ICMS tends to evoke smaller percepts than PNS^24^. As such, the level of annotation detail that is appropriate will likely also vary. Finally, technological literacy or familiarity will often vary across patients.

We have designed Sensory Survey 3D with many of these use-cases in mind. First, we include default models at various mesh resolutions. As the mesh resolution changes, so does the effective size of each annotatable face. In cases where high resolution is not prioritized or even feasible, low-face-count models can be used. Similarly, in the remeshing operation described for the generation of custom models, the target resolution can be customized to the users’ desire (**Supp Fig. 5**), and this operation can of course be applied to the default models. For participants with very limited mobility that must report sensations verbally or through a highly simplified interface, we also demonstrate the viability of simplified 2.5-dimensional models. These models are functionally equivalent to classical segment selectors or paper notation but can be easily analyzed programmatically and enable the annotation of oblique faces. Finally, we enable the definition of “default views” that allow users to cycle through predefined views such as the front or back of the hand while annotating, circumventing the need to pan across or orbit around the model. Particularly for participants with diminished dexterity, this can simplify interactions and streamline annotation.

## Conclusions

Paper-based and flattened maps of the body have been used in studies for decades but have obvious drawbacks. Sensory Survey 3D solves these problems by giving users an interactive tool for accurately and easily reporting sensations that can be adapted in a variety of ways. We show that in cases where projected fields are perpendicular to the user’s perspective, results are equivalent for 2D and 3D views. When body areas oblique to the user’s perspective are annotated, however, the results can be significantly skewed. We also demonstrate that custom body models can be generated and used for annotation and provide tools for standardizing analysis across models.

## Data Availability

The software package presented here is available on GitHub under the MIT license ([https://github.com/sensorimotor-bionics/SensorySurvey3D](https://github.com/sensorimotorbionics/SensorySurvey3D)). Example code for using the package and analyzing the data presented in this paper can also be found at the repository. Raw data used in this paper is stored at the Data Archive BRAIN Initiative (https://dabi.loni.usc.edu/dsi/XXXX).

## Abbreviations

2D: 2-dimensional
3D: 3-dimensional
BCI: brain computer interface
ICMS: intracortical microstimulation
PNS: peripheral nerve stimulation
